# Family Disclosure of a Cancer Diagnosis to Patients

**DOI:** 10.1001/jamanetworkopen.2026.9954

**Published:** 2026-04-30

**Authors:** Chenglei Hu, Hongyu Zhao, Yanhong Yan, Fang Fang, Chen Chen, Ping Yu, Jinrong Wei, Ericka Waidley, Josephine Hegarty, Pingting Zhu

**Affiliations:** 1 School of Nursing, Yangzhou University, Yangzhou, China; 2 Department of Nursing, Jiangsu Province Hospital, Nanjing, China; 3 Department of Nursing, Northern Jiangsu People’s Hospital Affiliated to Yangzhou University, Yangzhou, China; 4 Department of Cardiac Surgery, Nanjing Drum Tower Hospital, Affiliated Hospital of Nanjing University Medical School, Nanjing, China; 5 Department of Nursing, Affiliated Hospital of Yangzhou University, Yangzhou, China; 6 Department of Nursing, Yangzhou Hospital of Traditional Chinese Medicine, Yangzhou, China; 7 Linfield-Good Samaritan School of Nursing, Linfield College, Portland, Oregon; 8 School of Nursing and Midwifery, Brookfield Health Sciences Complex, University College Cork, Cork, Ireland

## Abstract

**Importance:**

In Chinese culture, families often take primary control over disclosing a cancer diagnosis, balancing a desire to protect patients’ emotional well-being with respect for their right to know. This dynamic can influence patients’ psychological health and overall treatment experience.

**Objective:**

To examine decision-making dynamics, communication strategies, and power structures involved in how Chinese families balance the delivery of information with patients’ psychological protection when disclosing a cancer diagnosis.

**Design, Setting, and Participants:**

This qualitative study used semistructured interviews with family members of adult patients with cancer in China. Purposive sampling ensured diversity in sex, age, and relationship to patients. Data collection and analysis were conducted simultaneously from June 1, 2024, to July 31, 2025, using thematic analysis.

**Main Outcomes and Measures:**

Key themes related to how families managed cancer diagnosis disclosure. Interviews elicited family members’ expectations, decision-making processes, and strategies for balancing protection and patient autonomy.

**Results:**

A total of 41 family members were interviewed (median [range] age, 38 [21-56] years; 24 females [58.5%]). Four themes were identified: (1) family negotiation and decision-making on diagnosis disclosure, (2) conflict and collaboration between families and health care professionals, (3) implementation of family-led initial disclosure strategies, and (4) dynamic adjustment of disclosure strategies with treatment progression. These themes illustrated how families continually balanced protection and autonomy in evolving clinical circumstances.

**Conclusions and Relevance:**

In this qualitative study, cancer diagnosis disclosure within Chinese families unfolded as a culturally embedded and fluid process, requiring ongoing negotiation and adjustment among family members. Given the continued prominence of families in medical decision-making in China, future interventions should aim to bridge the gap between family involvement in care and patient autonomy. While offering necessary family support, these strategies should also aim to steer the disclosure model toward one that elevates and protects the patient’s voice, thereby moving closer to internationally accepted ethical standards.

## Key Points

**Question:**

What are the experiences of Chinese family members in managing the disclosure of a cancer diagnosis to an adult patient?

**Findings:**

In this qualitative study including 41 family members of adult patients with cancer in China, 4 themes emerged in their descriptions of the disclosure process: collective family decision-making, negotiation with health care professionals, family-led initial disclosure, and continuous adjustment of information delivery throughout treatment.

**Meaning:**

These findings suggest that communication practices should accommodate culturally embedded family preferences while guiding a gradual transition to a patient-centered disclosure of cancer diagnosis in a Chinese context.

## Teaser

This qualitative study explores how Chinese family members manage the disclosure of a cancer diagnosis to adult patients.

## Introduction

The disclosure of a cancer diagnosis presents challenges for both the disclosing individual and the patient’s family, including emotional burden, role shifts, caregiving pressures, and communication breakdowns due to protective instincts.^1,2,3^ Navigating the challenges of disclosing a cancer diagnosis from an ethical perspective involves balancing key ethical principles, such as autonomy, beneficence, nonmaleficence, and justice, while being sensitive to the patient’s needs, the complex dynamics of family, and the cultural context.^4,5,6^ This can create a conflict between respect for cultural norms, for example, a family’s preference for receiving the diagnosis first or a request that the patient not be informed, and the patient’s right to know.^7,8^

There is no consensus on whether or how to disclose news of a cancer diagnosis to patients.^9,10,11^ In Western health care systems, the principle of patient autonomy is emphasized, and physicians typically inform patients of their diagnosis directly.^4^ In contrast, in many Asian countries, such as China,^12^ Pakistan,^13,14^ and Lebanon,^15^ many patients with cancer are unaware of their diagnosis. Physicians are more willing to disclose the cancer diagnosis to the patients’ families, and it is up to the family members to decide whether to inform patients.^16,17^ This is especially true in China; as the country with the highest number of new cancer cases globally,^18^ families play a core role in medical decision-making.^19^ A cross-sectional study of 190 medical staff in Nanjing, China, found that 70.5% (n = 134) supported direct disclosure of a cancer diagnosis to patients, but in practice, 72.1% (n = 137) delegated this decision to patients’ families.^20^ This phenomenon is related to the traditional Confucian culture in China, which emphasizes family harmony, responsibility, and collective decision-making.^21,22^ In addition to cultural factors, physicians’ tendency to disclose diagnoses to family members is reinforced by limited public awareness of patient autonomy, resource constraints, high treatment costs, and a desire to mitigate potential conflict.^23^

Current domestic and international research mainly classifies family disclosures regarding cancer into 3 types: full disclosure, partial disclosure, and avoidance of disclosure.^24,25,26^ A meta-analysis encompassing 22 studies revealed that nearly 89.6% of patients with cancer in China (n = 1692 in 6 studies;95% CI, 80.7%-94.7%; *I*^2^ = 0.955) wanted to know their diagnosis, but 50% of their family members (n = 1156 in 5 studies; 95% CI, 32.8%-67.2%; *I*^2^ = 0.966) chose to conceal the diagnosis.^27^ This finding indicates a discrepancy between patients’ and their family members’ preferences regarding diagnosis disclosure.^17^ However, the disclosure of a cancer diagnosis is a complex and dynamic process rather than a single moment.^28,29^ While research on diagnostic disclosure has been increasing globally, in China, the process is often shaped by complex family decision-making, a dynamic that remains insufficiently explored.

The purpose of this study was to explore the disclosure experiences of family members of adult patients in China, in terms of their receipt of the cancer diagnosis and continued sharing of cancer treatment–related information, their associated interactions with health care professionals, and their sharing of information with one another and the patients with cancer. We sought opportunities to explore current disclosure processes, with the aim of informing recommendations to move toward a patient-centered disclosure of cancer diagnosis.

## Methods

This qualitative study was approved by the Ethics Committee of the School of Nursing, Yangzhou University. Consent from all participants was obtained prior to the interviews. The study adhered to the Consolidated Criteria for Reporting Qualitative Research (COREQ) guideline.^30,31^

### Design and Settings

This study was part of a larger mixed-methods study, the Family Cancer Disclosure Study of Chinese Patients With Cancer, focusing on the qualitative data. Participants were adult family members of patients with cancer, recruited from both the inpatient and outpatient oncology departments of 5 tertiary hospitals. The hospitals were located in Jiangsu Province, China, an economically developed coastal region with nationally leading health care infrastructure and medical standards. We used purposive sampling, seeking a range of family member characteristics (sex, age, and relationship to patients). Additionally, we considered patient characteristics, such as sex, age, marital status, cancer type, cancer stage, and time since cancer diagnosis, to capture a range of perspectives on disclosure.

### Data Collection

From June 1, 2024, to July 31, 2025, 3 researchers (C.H., P.Z., and H.Z.) developed a semistructured interview guideline (eAppendix in Supplement 1), which was informed by a literature review and expert consultation. A researcher (P.Z.) provided study details to the head nurses in the oncology departments, who helped researchers invite eligible families to participate. Face-to-face interviews were conducted in quiet rooms at the hospitals, while remote interviews were held via an encrypted video conferencing platform (Tencent Meeting; Tencent Cloud). Written informed consent was obtained for face-to-face interviews, and digital consent was recorded for remote interviews.

All interviews were conducted in Mandarin Chinese by researchers (C.H. and H.Z.) who had received training during their graduate studies in qualitative research methods from 2 nursing faculty members with doctoral degrees. With participants’ permission, audio recordings and handwritten notes were taken. Nonverbal cues (eg, facial expressions and body language) were noted to help contextualize the participants’ narratives but were not formally coded or systematically analyzed as study data. Participants were also required to complete an online demographic survey.

Purposive sampling was used, and a semistructured interview guide, continuously refined during data collection, was used in conjunction with strategic follow-up questions to encourage participants’ in-depth reflection and active narration, ensuring data sufficiency.^32^ Interview content was also assessed throughout data collection to ensure sufficiency for the identification of themes and subthemes.^33^ A total of 46 people were invited to participate; 4 refused, 1 could not be contacted, and 41 agreed to participate. Interviews were conducted in person with 32 participants and remotely with 9. Interview durations ranged from 63 to 172 minutes.

### Data Analysis

The recorded interviews were transcribed and deidentified by researchers (C.H. and H.Z.). The transcriptions were checked for accuracy by a third researcher (P.Z.). Two researchers (C.H. and P.Z.) independently conducted thematic analysis^34^ using the 6-phase framework of Braun and Clarke.^33^ They familiarized themselves with the data, generated initial codes in NVivo version 12 (Lumivero), and collated them into potential themes. These themes were reviewed, refined, and named through discussions with the entire research team. Throughout the process, the research team held regular consensus meetings to discuss emerging concepts and resolve coding discrepancies.

All interviews, transcripts, and analyses were in Mandarin Chinese. The themes and quotations were translated into English by researchers (C.H. and H.Z.). An international expert in qualitative research (J.H.) reviewed the data linked to each theme to assess whether it genuinely supported the findings and evaluated how well the themes fit within the context of the dataset. A member of the research team (Y.Y.) cross-checked the Chinese and English versions for consistency. We ensured trustworthiness through member checking and maintaining a clear audit trail. Participants reviewed the preliminary results to assess accuracy.

## Results

A total of 41 family members were interviewed (median [range] age, 38 [21-56] years; 24 females [58.5%] and 17 males [41.5%]). Most participants were spouses of patients (22 [53.7%]), followed by sons or daughters (12 [29.3%]). The characteristics of the participants are presented in Table 1. Table 2 reports the 4 themes that emerged from the interviews alongside their corresponding subthemes, and Table 3 lists representative quotations for each.

**Table 1.** Characteristics of Participants and Associated Patients With Cancer

Characteristic	No. (%)^a^
Participants	
Sex	
Female	24 (58.5)
Male	17 (41.5)
Age, median (range), y	38 (21-56)
Relationship to patient	
Spouse	22 (53.7)
Son or daughter	12 (29.3)
Daughter-in-law or son-in-law	2 (4.9)
Father or mother	5 (12.2)
Patients	
Sex	
Female	22 (53.7)
Male	19 (46.3)
Age, median (range), y	44 (20-63)
Marital status	
Married	34 (82.9)
Single	7 (17.1)
Cancer site	
Lung	5 (12.2)
Colorectal	9 (22.0)
Gastric	4 (9.8)
Thyroid	7 (17.1)
Breast	6 (14.6)
Liver	4 (9.8)
Prostate	3 (7.3)
Cervical	3 (7.3)
Cancer stage	
I	6 (14.6)
II	9 (22.0)
III	10 (24.4)
IV	7 (17.1)
Unknown	9 (22.0)
Time since cancer diagnosis, mo	
<1	13 (31.7)
1 to <6	10 (24.4)
6 to <12	7 (17.1)
≥12	11 (26.8)
Hospital for diagnosis and treatment	
A	7 (17.1)
B	9 (22.0)
C	8 (19.5)
D	7 (17.1)
E	10 (24.4)

^a^ Percentages may not sum to 100 due to rounding.

**Table 2.** Themes and Subthemes

Theme	Subtheme
Family negotiation and decision-making on diagnosis disclosure	Seeking and sharing of cancer information within the family Comprehensive family assessment of the patient’s perceived capacity to cope Deference to perceived family authority figures and hierarchical decision-making
Conflict and collaboration between families and health care professionals	Conflict between physician’s support for informing the patient and family’s protective concealment Conflict over the right time to inform the patient of cancer diagnosis Negotiation of cancer disclosure boundaries and communication messages
Implementation of family-led initial disclosure strategies	Concealment of pessimistic emotions to support the patient Alternative language for cancer-related medical terms Hope management for patients with cancer
Dynamic adjustment of disclosure strategies with treatment progression	Reevaluation of disclosure strategy triggered by patient’s suspicion Progressive information release under conditions of perceived therapeutic benefit Revisiting of family decisions triggered by cancer progression or recurrence Family decision-making conflicts arising from changes in treatment goals at the end of life

**Table 3.** Subthemes and Illustrative Quotations

Subtheme	Illustrative quotation
Seeking and sharing of cancer information within the family	“When the doctor told me that my mother was diagnosed with breast cancer, I found it hard to accept this harsh reality at first. After gradually calming down, I immediately shared the devastating news with my brother and discussed with him whether he knew any friends or relatives in the medical field who could help us better understand the condition and guide us on the next steps for treatment and care.” “I remembered a relative who mentioned that his father had lung cancer, so I called him to ask for advice while I also searched the internet for information.” “My family was a single-parent family. In addition to my sick father, I also had a younger brother in high school. When my father was hospitalized, I had no one to talk to, and I was also afraid to tell anyone, including my girlfriend. I searched for information on my own and tried to find the best medical specialists to help with his treatment.”
Comprehensive family assessment of the patient’s perceived capacity to cope	“My mother’s health had already been compromised by cancer, and she suffered from mild depression. If she were informed of her cancer diagnosis, she would experience profound fear.” “My child might even be more knowledgeable than I was. Nowadays, college students could often figure out what illness they had by searching online. And since thyroid cancer could be treated with surgery, we decided to tell him about the diagnosis.” “My husband was a high school teacher, usually very busy and serious about his work. I felt it was hard to keep it from him because he was the one who made the decisions for the family and had a respected position at school. Besides, I knew that being ill would definitely affect his work, so I did not want to hide the truth from him.”
Deference to perceived family authority figures and hierarchical decision-making	“In our family, my eldest uncle held the final say, and he was absolutely opposed to disclosing the diagnosis to Grandma. His youngest brother, who bore the majority of the treatment costs, agreed with him. When they were aligned, the rest of us followed.” “A disagreement arose among my siblings and me regarding whether to disclose my mother’s cancer diagnosis to her. Ultimately, we deferred to our eldest sister’s judgment and decided to inform her. This decision was reached considering that I was still pursuing my education, my brother was employed in a different city, and our father abstained from voicing a strong opinion, leaving us with the perception that our eldest sister possessed the most profound understanding of our mother.” “I wanted to tell my mother slowly that she had cancer, but my father wouldn’t let me. He said it would upset her too much, so I had to hold back.”
Conflict between physician’s support for informing the patient and family’s protective concealment	“The doctor kept telling us that the patient had a right to know the truth, but we didn’t dare tell him the diagnosis out of fear that it would devastate him. I felt that at that time, we just wanted to hold off and wait to see the results of the treatment before deciding whether to tell my dad about the cancer diagnosis.” “The doctor wanted the patient to know earlier, but we felt that telling him at that point would only cause more distress, and the mental burden would be too great.”
Conflict over the right time to inform the patient of cancer diagnosis	“The doctor wanted to tell the patient before surgery, but we insisted on waiting until we got home after discharge.” “The doctor told me that it was best to tell the patient, but I felt it was not the right time. I wanted to see the results of the treatment and let her know when it was good.”
Negotiation of cancer disclosure boundaries and communication messages	“My dad has been diagnosed with terminal cancer and has about 6 months to live. We told him that there was still hope for treatment and asked the physician to share more successful treatment stories with him.” “The doctors discussed with us the extent of information to disclose about the diagnosis while we provided them with updates on my father’s condition at home. Through this collaboration, we agreed on the most appropriate way to communicate with him. A key part of our strategy was to ensure complete consistency between the medical team’s messages and our family’s conversations with him, to avoid any conflicting information that could have increase his anxiety or sown doubt.”
Concealment of pessimistic emotions to support the patient	“As soon as the diagnosis was delivered, I told my children not to ask your mother too much and to be as positive as possible around her.” “Holding back my emotions, I told my mother that she had a lump in her stomach and promised a recovery after surgery. My sister, too distressed to face the conversation and fearing her tears would give everything away, excused herself to ‘buy something,’ leaving me alone to explain.”
Alternative language for cancer-related medical terms	“We told him it was just a lump and specially asked the doctor to explain it that way, as he trusted the doctor more. Our aim was to keep the message simple and consistent, thereby avoiding unnecessary anxiety.” “We described chemotherapy as medicine to help the body recover, avoiding terms like *cancer treatment*.”
Hope management for patients with cancer	“Our family believed that getting him to be willing to cooperate with the treatment was the most important thing, because we feared that fully disclosing the diagnosis would lead him to despair.” “We selectively shared the diagnosis with him, focusing on and emphasizing positive aspects such as the possibility of a cure and a timeline for returning home.” “Before my father was diagnosed with cancer, my family didn’t believe Buddhism. But I noticed that when I took him to worship the Buddha, his mood seemed to improve, and he perhaps became a bit more hopeful about fighting the disease.”
Reevaluation of disclosure strategy triggered by patient’s suspicion	“After I met with the doctor to discuss the surgical plan, my mother went into the consultation room herself and asked the doctor to tell her what was wrong with her. The doctor immediately called me in. Faced with this, I had to confer with the doctor and other family members on how to disclose more information while calming my mother down.” “I noticed my father suddenly became very quiet. When I gently tried to talk to him, he didn’t respond. Later, he told me he had searched online and suspected his symptoms might be cancer. His suspicion made us uncertain whether to directly tell him it was cancer or to use more indirect explanations. We also thought it necessary for the doctor to discuss his condition again to address his doubts and provide reassurance.”
Progressive information release under conditions of perceived therapeutic benefit	“I found that my mom’s symptoms were under control and her mood was stable. After consulting the doctor, I decided to disclose the truth gradually. I first asked her, hypothetically, if life might not be long, could she accept it? She even joked, ‘Of course, otherwise the earth would be unlivable.’ Encouraged by her calmness, I continued to tell her the diagnosis step by step, so she could process the information without feeling overwhelmed.” “We told our mother about the cancer diagnosis when she went home to recuperate after the surgery. At this point, the surgery was successful, the condition was relatively stable, and she would not be too afraid. Otherwise, when she finds out for herself, she’ll think more and argue with us.”
Revisiting of family decisions triggered by cancer progression or recurrence	“My wife suddenly began vomiting. After she received medical treatment, the doctor informed us that all her indicators were abnormal. I panicked and immediately called her brother to discuss what to do. We had never disclosed her cancer diagnosis because she had previously been stable, and we saw no need to alarm her. Now the situation had suddenly taken a turn for the worse, I felt even more uncertain and afraid to tell her. I was terrified that the shock of the news might cause her condition to deteriorate faster.” “My mother suddenly sat down on the ground, crying out in pain. We rushed her to the hospital for a checkup, where we discovered the cancer had in fact recurred 3 months prior. I immediately called my brother to discuss what to do. The doctor said we needed to change the treatment plan. I was worried that, compared to the first diagnosis, the new treatment plan would make her more suspicious and lead to more questions.”
Family decision-making conflicts arising from changes in treatment goals at the end of life	“From my father’s diagnosis to his passing was only 10 months. We never dared to tell him what the doctor had said. The struggle became unbearable when we learned his time was less than 3 months and there was no cure. I was torn: should I tell him the truth and bring him home to spend his final months with the family, or cling to the hope of treatment and pray for a miracle? The treatment costs were enormous, and we were also responsible for other family members, especially the children. I tried many times to bring it up with my father, but faced with my sister’s firm insistence on continuing treatment, I ultimately couldn’t go through with it.” “The doctor told me that, given my father’s condition with advanced liver cancer, there was no effective treatment anymore, and they could not say how long my father might live. My brother asked me if we should tell him the full truth and honor his wishes. I kept wondering if it would be unfilial to give up so early. Although I struggled a lot, I finally agreed. We took my father home to spend the New Year together and gradually began to reveal the truth to him. After that, we never went back to the hospital.”

### Theme 1: Family Negotiation and Decision-Making on Diagnosis Disclosure

#### Seeking and Sharing of Cancer Information Within the Family

Close collaboration among family members was considered crucial for gathering and synthesizing cancer-related information to ensure optimal care for the patient. In addition to consulting health care professionals, most family members sought information online and advice from individuals who had cared for patients with cancer. A few participants, however, chose to shoulder the burden alone, citing the youth, advanced age, or emotional fragility of other family members.

#### Comprehensive Family Assessment of the Patient’s Perceived Capacity to Cope

Family members assessed the patient’s cognitive capacity and emotional resilience when considering disclosure. For example, 1 participant stated, “My mother’s health had already been compromised by cancer.… If she were informed of her cancer diagnosis, she would experience profound fear.” In addition, some participants also considered the patient’s age, education, work role, and social status.

#### Deference to Perceived Family Authority Figures and Hierarchical Decision-Making

Family decision-making frequently involved internal negotiation. Final decisions were often influenced by authority figures, such as the primary caregiver, financial provider, family elder, or eldest child. For instance, 1 participant said, “I wanted to tell my mother slowly…but my father wouldn’t let me…so I had to hold back.”

### Theme 2: Conflict and Collaboration Between Families and Health Care Professionals

#### Conflict Between Physician’s Support for Informing the Patient and Family’s Protective Concealment

Family members reported that physicians underscored the importance of disclosing the diagnosis to enable the patient’s involvement in treatment decisions. Nevertheless, particularly in advanced cancer, many still preferred to conceal the diagnosis as a way to protect the patient’s psychological well-being. This protective stance was common, as reflected in 1 participant’s account: “The doctor kept telling us that the patient had a right to know the truth, but we didn’t dare tell him the diagnosis out of fear that it would devastate him.”

#### Conflict Over the Right Time to Inform the Patient of Cancer Diagnosis

Family members reported that while they often sought to delay disclosure, physicians typically provided some diagnostic information early in the clinical encounter. One family member said, “The doctor wanted to tell the patient before surgery, but we insisted on waiting until we got home after discharge.” In practice, however, most participants indicated that physicians generally respected the choice made by the family.

#### Negotiation of Cancer Disclosure Boundaries and the Harmonization of Communication Messages

Family members discussed with physicians which information should be shared with the patient to prevent excessive psychological distress. One participant said, “My dad has been diagnosed with terminal cancer and has about 6 months to live. We told him that there was still hope for treatment and asked the physician to share more successful treatment stories with him.” Continuous communication and negotiation helped ensure message consistency.

### Theme 3: Implementation of Family-Led Initial Disclosure Strategies

#### Concealment of Pessimistic Emotions to Support the Patient

Family members often suppressed their own grief to present a supportive and optimistic attitude, aiming to provide warmth and strength to the patient. One participant said, “As soon as the diagnosis was delivered, I told my children not to ask your mother too much and to be as positive as possible around her.” This conscious effort to maintain positivity, however, came at a personal cost.

#### Alternative Language for Cancer-Related Medical Terms

Families often used euphemistic language, such as “a lump that needs further examination” or “inflammation that requires monitoring,” when discussing the diagnosis with the patient. They believed such descriptions were more motivating and helped improve patient adherence to treatment.

#### Hope Management for Patients With Cancer

To sustain hope, some families strategically emphasized the more hopeful aspects of the diagnosis, such as treatment efficacy and potential favorable outcomes, rather than disclosing the full diagnosis. In addition to these informational strategies, a few participants engaged in religious practices (eg, worshipping Buddha) as a form of spiritual coping with the same goal of preserving hope.

### Theme 4: Dynamic Adjustment of Disclosure Strategies With Treatment Progression

#### Reevaluation of Disclosure Strategy Triggered by Patient’s Suspicion

Some family members noted that concealment could arouse the patient’s suspicion, prompting them to seek information independently. Once family members became aware of this, they had to reevaluate their approach, deciding whether to continue working with health care professionals as they had before or shift toward a more controlled and partial disclosure.

#### Progressive Information Released Under Conditions of Perceived Therapeutic Benefit

Families often aligned the gradual disclosure of the diagnosis with observed clinical improvement in the patient’s condition. One participant shared, “We told our mother about the cancer diagnosis when she went home to recuperate after the surgery. At this point, the surgery was successful, the condition was relatively stable, and she would not be too afraid.” This strategy increased patient cooperation and reduced family conflict.

#### Revisiting of Family Decisions Triggered by Cancer Progression or Recurrence

The progression or recurrence of cancer often required adjustments to the patient’s treatment and care plans. Participants explained that providing a plausible explanation for new treatments or additional care without revealing the diagnosis became increasingly difficult, heightening their anxiety about the concealment unraveling.

#### Family Decision-Making Conflicts Arising From Changes in Treatment Goals at the End of Life

As the patient’s condition reached the terminal stage, family members faced the dilemma of whether to continue treatment or disclose the full truth. Conflicts often arose between family members, especially when discontinuing treatment conflicted with the cultural value of filial piety.

## Discussion

This qualitative study assessed the perspective of patients’ family members, exploring the intricate process of disclosing a cancer diagnosis to patients and identifying 4 key themes. In China, family members usually made disclosure decision-making within the family after seeking cancer information and assessing the patient’s status. Families accepted information provided by physicians, but they also turned to their extended community or online sources to supplement their knowledge.^35^ In the context of supporting decision-making within families, health care professionals need to provide clear and accurate cancer information to key family members,^36^ when they first inform the family of a cancer diagnosis pertaining to a loved one.

Our study found family members typically engaged in multiple rounds of communication and negotiation with physicians to balance family opinions with professional judgment after reaching a preliminary disclosure decision within the family. In the context of strained relations between physicians and patients in China,^37,38^ when family members request the withholding of a diagnosis on the grounds of the patient’s best interest, physicians often chose to comply, despite feeling conflicted and confused.^39^ Family members believe that withholding such a diagnosis aligns with the Confucian value of *He* (harmony), which emphasizes protecting the vulnerable and maintaining family unity.^21,40^ However, while Confucianism prioritizes care, it also upholds *Yi* (righteousness) and *Xin* (trustworthiness). Withholding the diagnosis also conflicts with such virtues.^41,42^ Previous studies have suggested strategies for addressing family requests for nondisclosure (eg, acknowledging requests, seeking common goals, and outlining harms), although these approaches still lack strong empirical support.^43,44,45^

Cancer now often has a much better prognosis than in the past, but the diagnosis can still evoke fear and is commonly perceived as hopeless or a synonym for a death sentence.^46,47,48,49^ At the initial disclosure stage, family members consistently enacted 3 protective practices: concealing their pessimistic emotions, using softened or substitute statements for medical facts, and actively maintaining patient hope.^50,51^ These findings are consistent with existing literature showing that family members enter into a so-called conspiracy of silence with physicians to avoid the emotional burden of direct disclosure of cancer.^52,53^ However, hope can be preserved through means other than concealment. Supportive psychological interventions, such as meaning therapy, dignity therapy, mindfulness-based interventions, and life review, have been shown to significantly alleviate depression, anxiety, and hopelessness, while improving quality of life and fostering optimism in patients with cancer.^54,55^

Maintaining concealment of a cancer diagnosis was often unfeasible, as most patients proactively sought information and discovered the truth due to suspicions regarding their own health status and their families’ behavior.^56,57^ Rather than adhering rigidly to family requests for nondisclosure, clinicians should attend closely to indications that a patient may already suspect the truth, allowing clinicians to provide support that lessens isolation and aligns with the patient’s lived awareness. Moreover, a systematic review and meta-analysis of 23 studies concluded that disclosing a cancer diagnosis did not adversely affect patients’ overall quality of life and was associated with substantially higher vitality in fully informed patients.^58^ In the terminal stage of cancer, Chinese families often faced a conflict between extending survival and preserving quality of life, compounded by economic burdens and the cultural norms of filial piety. Disclosing the cancer diagnosis enabled patients with cancer to arrange end-of-life care according to their preferences, with autonomous decisions on matters such as prioritizing quality of life and choosing a home death.^59^

Our study provides key insights into current cancer diagnosis disclosure practices, emphasizing the need for clear and accurate information to support family decision-making. Clinicians in China should be trained to balance family involvement while gradually centering on the patient’s voice in the disclosure process. Future research should explore how these disclosure practices evolve across illness trajectories and in different care settings, particularly in family-centric cultures. Research in other Asian countries and among Asian diaspora communities could offer valuable comparisons and inform culturally sensitive strategies. Guidance for clinicians on implementing a patient-centered and family-informed approach to diagnosis disclosure should be cocreated with health care professionals, patients, and families in a Chinese context. Mixed-methods research could clarify how common different disclosure patterns are and their association with outcomes such as distress or decisional conflict. Conceptual and bioethical analyses could support the development of context-sensitive disclosure frameworks.

### Limitations

This study has limitations. First, it was conducted only in Jiangsu Province, China, so its findings may not apply to other countries or cultural contexts. Second, while purposive sampling was used to enhance diversity, voluntary participation may have led to selection bias, with potential underrepresentation of family members who faced difficulties or were reluctant to share their experiences. Lastly, the patient age range of up to 63 years may not adequately capture the higher levels of frailty, increased caregiving needs, and more complex proxy decision-making typical among adults aged 65 years or older.

## Conclusions

This study used qualitative methods to explore the decision-making processes and experiences of family members of patients with cancer regarding diagnosis disclosure in China. Based on these findings, health care professionals could be encouraged to support the structured involvement of families while progressively centering on patients’ voices by using a phased disclosure strategy to guide family members in choosing the appropriate time and method to disclose diagnosis information to patients. We advocate for transitioning to a patient-first disclosure model as a preferred approach in future practice. The goal should be to foster informed and shared decision-making among health care professionals, patients with cancer, and their family members, ultimately leading to care that aligns with patient needs and improves satisfaction with disclosure decision-making processes.

## Notes

Supplement 1. **eAppendix.** Semistructured Interview Guideline

Supplement 2.
Data Sharing Statement
