# Supplementary material for: Family Disclosure of a Cancer Diagnosis to Patients
Source: JAMA Netw Open. 2026 Apr 30;9(4):e269954. doi: 10.1001/jamanetworkopen.2026.9954 (PMC13133687; doi:10.1001/jamanetworkopen.2026.9954)
Supplement: Supplement 1. — eAppendix. Semistructured Interview Guideline [file jamanetwopen-e269954-s001.pdf]

Supplemental Online Content

Hu C, Zhao H, Yan Y, et al. Family disclosure of a cancer diagnosis to patients. *JAMA Netw Open*. 2026;9(4):e269954. doi:10.1001/jamanetworkopen.2026.9954

eAppendix. Semistructured Interview Guideline

This supplemental material has been provided by the authors to give readers additional information about their work.

eAppendix

Semistructured Interview Guideline

1. How did the doctor initially inform you about the patient's cancer diagnosis?
2. How did you decide whether to disclose the true diagnosis to the patient?
3. What were the most influential factors in your decision-making process? (e.g., family opinions/ cultural beliefs/patient's personality)
4. What specific approach did you ultimately use to inform the patient?
5. How did the patient react after learning the diagnosis, and how did this influence your subsequent disclosure methods?
6. What roles did other family members play in the decision-making process?
7. What was the greatest challenge you encountered throughout this process?
8. Did your approach to disclosure change as treatment/cancer journey progressed?
9. Looking back, would you make different choices now?
