# Supplementary material for: Family Disclosure of a Cancer Diagnosis to Patients
Source: JAMA Netw Open. 2026 Apr 30;9(4):e269954. doi: 10.1001/jamanetworkopen.2026.9954 (PMC13133687; doi:10.1001/jamanetworkopen.2026.9954)
Supplement: Supplement 2. — Data Sharing Statement [file jamanetwopen-e269954-s002.pdf]

# Data Sharing Statement

Hu. Family Disclosure of a Cancer Diagnosis to Patients. *JAMA Netw Open*. Published April 30, 2026. doi:10.1001/jamanetworkopen.2026.9954

**Data**
**Data available:** No

**Additional Information**
**Explanation for why data not available:** Given the nature of qualitative research, it is not feasible to archive complete anonymization of interview data. Participants may still be identifiable based on the content of their responses, even after removing names, institutions, and other direct identifiers. Therefore, to protect participant confidentiality, we are unable to publicly share the interview transcripts. Researchers interested in potential collaboration using this dataset are welcome to contact me (C.H.) to discuss possibilities for further joint research.
